# GPU-accelerated ray-casting for 3D fiber orientation analysis

**DOI:** 10.1371/journal.pone.0236420

**Published:** 2020-07-29

**Authors:** Roman Shkarin, Svetlana Shkarina, Venera Weinhardt, Roman A. Surmenev, Maria A. Surmeneva, Andrei Shkarin, Tilo Baumbach, Ralf Mikut

**Affiliations:** 1 Laboratory for Applications of Synchrotron Radiation, Karlsruhe Institute of Technology, Karlsruhe, Germany; 2 Institute for Automation and Applied Computer Science, Karlsruhe Institute of Technology, Karlsruhe, Germany; 3 Physical Materials Science and Composite Materials Centre, Research School of Chemistry & Applied Biomedical Sciences, National Research Tomsk Polytechnic University, Tomsk, Russia; 4 Institute for Photon Science and Synchrotron Radiation, Karlsruhe Institute of Technology, Eggenstein-Leopoldshafen, Germany; 5 Centre for Organismal Studies, COS, Heidelberg University, Heidelberg, Germany; Hong Kong Polytechnic University, HONG KONG

## Abstract

Orientation analysis of fibers is widely applied in the fields of medical, material and life sciences. The orientation information allows predicting properties and behavior of materials to validate and guide a fabrication process of materials with controlled fiber orientation. Meanwhile, development of detector systems for high-resolution non-invasive 3D imaging techniques led to a significant increase in the amount of generated data per a sample up to dozens of gigabytes. Though plenty of 3D orientation estimation algorithms were developed in recent years, neither of them can process large datasets in a reasonable amount of time. This fact complicates the further analysis and makes impossible fast feedback to adjust fabrication parameters. In this work, we present a new method for quantifying the 3D orientation of fibers. The GPU implementation of the proposed method surpasses another popular method for 3D orientation analysis regarding accuracy and speed. The validation of both methods was performed on a synthetic dataset with varying parameters of fibers. Moreover, the proposed method was applied to perform orientation analysis of scaffolds with different fibrous micro-architecture studied with the synchrotron μCT imaging setup. Each acquired dataset of size 600x600x450 voxels was analyzed in less 2 minutes using standard PC equipped with a single GPU.

## Introduction

Quantification analysis of fiber orientation is frequently required in the fields of medical, material and life sciences. The orientation allows to predict properties of materials reinforced with fibers and regeneration speed of tissues with integrated scaffolds, validate and guide a fabrication process of scaffolds with controlled fiber orientation.

There is a vast number of approaches for quantification of fiber orientation. The application of each approach greatly depends on an imaging method and hardware that dictates data dimensionality, dynamic range, and spatial resolution expressing a minimal resolvable size of structures.

Over the last decades, several approaches for two-dimensional (2D) data analysis emerged. The Hough transform was utilized for analysis of collagen fibers [1], electrospun polyacrylonitrile fibers [2], and alignment quantification of structures in textile composites [3]. The approach using intensity gradient and its magnitudes allowed to determine the orientation at every pixel of images acquired from cytoskeletal fibers [4], myofiber disarray [5], fibers in human ligament fibroblast [6], unidirectional fiber reinforced polymers [7], and collagen fibers [8]. The methods involving the analysis of spatial frequency components of the 2D Fourier spectrum enabled to reveal a global orientation of presented structures. They were applied to quantify direction of nanofibrous and nonwovens layers of textile materials [9,10], amorphous cast iron fibers [11], actin fibers and myofibroblasts [12], scleral fibers in normal rat eyes [13], fibers in electrospun materials [14–16], collagen fibers [17,18], and fibroblast proliferation [19]. Another way to utilize the 2D Fourier spectrum is to fit a line into the thresholded spectrum to infer orientation from a line slope [20] to estimate the direction of α-actin fibers [21]. Also, the Radon transform was employed for quantification of fiber alignment [22].

Recent advances in the development of micro-computed tomography (μCT) and confocal laser scanning microscopy (CLSM) enable to reveal a three-dimensional (3D) microstructural information of a sample. Acquisition of X-ray projections of the sample from a range of angles allows reconstructing the stack of cross-sections of the sample with the help of tomographic reconstruction algorithms [23,24]. CLSM allows obtaining successive sample optical sections at different depth levels which provide a 3D representation of the sample. These 3D imaging methods in conjunction with subsequent processing techniques produce 3D datasets represented as stacks of 2D images. To quantify the orientation of fiber structures in a 3D space, various approaches analyzing data in a neighborhood of each voxel were proposed.

Estimation of the second-order structure tensor at a voxel neighborhood allows computing eigenvalues and eigenvectors of the region. The smallest eigenvalue corresponds to the eigenvector pointing towards a direction of a primary orientation of the structures within this region. The approach is based on a computation of a structure tensor at every point of a medial axis of segmented fibers in reinforced composite, nonwoven fabrics, and fibrous porous networks, produced by a morphological thinning or trajectory tracing algorithm [25–32]. A similar approach was suggested for investigation of scaffold organization of the engineered heart valve tissue for post- and pre-implants [33]. For the quantification of electrospun fiber mats organization, a tensor calculation at every voxel of segmented fibers with maintaining a neighborhood radius larger than maximal fiber diameter was proposed in [34]. Another approach measures orientation of fibers in planar sections of reinforced composite materials by detecting elliptical and non-elliptical footprints to derive a 3D structure tensor [35–38]. Calculation of 3D inertia moments of boundary points of the segmented fiber was proposed to estimate the orientation of the collagen fibers [39,40]. The analysis of the 3D Fourier spectrum allowed to determine orientation in collagen-based tissues within local 3D windows by performing correlation with 3D orientation fiber banks [41]. The approach based on parameter-tuning of an anisotropic 3D Gaussian filter generates a filter of a prolate spheroid shape to find its maximum response at every voxel, that corresponds to a primary orientation when a filter direction coincides with the direction of a structure in that voxel [42]. The algorithm based on a weighted sum of vectors pointing towards each element in a neighborhood from the central element was proposed and used for investigation of an organization of fiber structures in biological tissues [43–45].

Development of modern detectors has led to an increase in the amount of generated data per sample and required computation time. In a case of 2D orientation analysis approaches, it did not make an issue, since modern central processing units (CPUs) can easily handle 2D problems in a reasonable amount of time. On the other hand, it increased required computation resources for 3D approaches to be executed in reasonable time frames. Mentioned approaches provide high-quality analysis, but unfortunately, current 3D approaches are not rapid enough for processing of large data. Moreover, up to the author's knowledge, there is no reported progress on the implementation of these approaches for modern graphics processing units (GPUs).

The conventional workflow for orientation analysis of μCT datasets is composed of several stages: pre-processing, segmentation, a medial axis extraction, and orientation quantification. Diverse points of views to this workflow were presented in a range of works devoted to the analysis of fibrous structures in life and material sciences [8,29,46,47].

The quality of the acquired μCT data varies and depends on parameters of an imaging method, an acquisition device, a sample preparation protocol and nature of a sample. Therefore, the pre-processing stage can partially correct weak noise with non-linear filters like median, bilateral or other edge preserving filters. On the other hand, the low contrast data, or contaminated with beam hardening, streak or ring artifacts [48,49] are very challenging and required specifically tailored procedures to correct them.

The pre-processed data should be segmented to proceed with the further analysis. Despite the diversity of segmentation techniques, their applicability depends on the nature of the data. When fibers are made of homogeneous material and the data possess high contrast, then simple histogram-based methods might be applied. Otherwise, more sophisticated approaches involving texture analysis and machine learning are better suited.

A medial axis or a skeleton is a centerline extracted from isolated binary regions of the segmented data. There are many algorithms for deriving the medial axis, some of them consider crossing or touching regions [50–53].

Based on the above, here we propose a novel 3D approach for quantification of fiber orientation based on the ray-casting idea. We have compared the proposed method with the method based on the second-order structure tensor in terms of accuracy and throughput. The accuracy was verified on a synthetic dataset with varying fiber density, alignment, and diameter. The throughput was measured for several implementations tailored to computational environments consisting of CPUs and GPU. Finally, the proposed approach was successfully applied for the analysis of real-world datasets acquired with the synchrotron μCT imaging setup.

## Materials and methods

### Ray-casting algorithm

**Fig 1:**
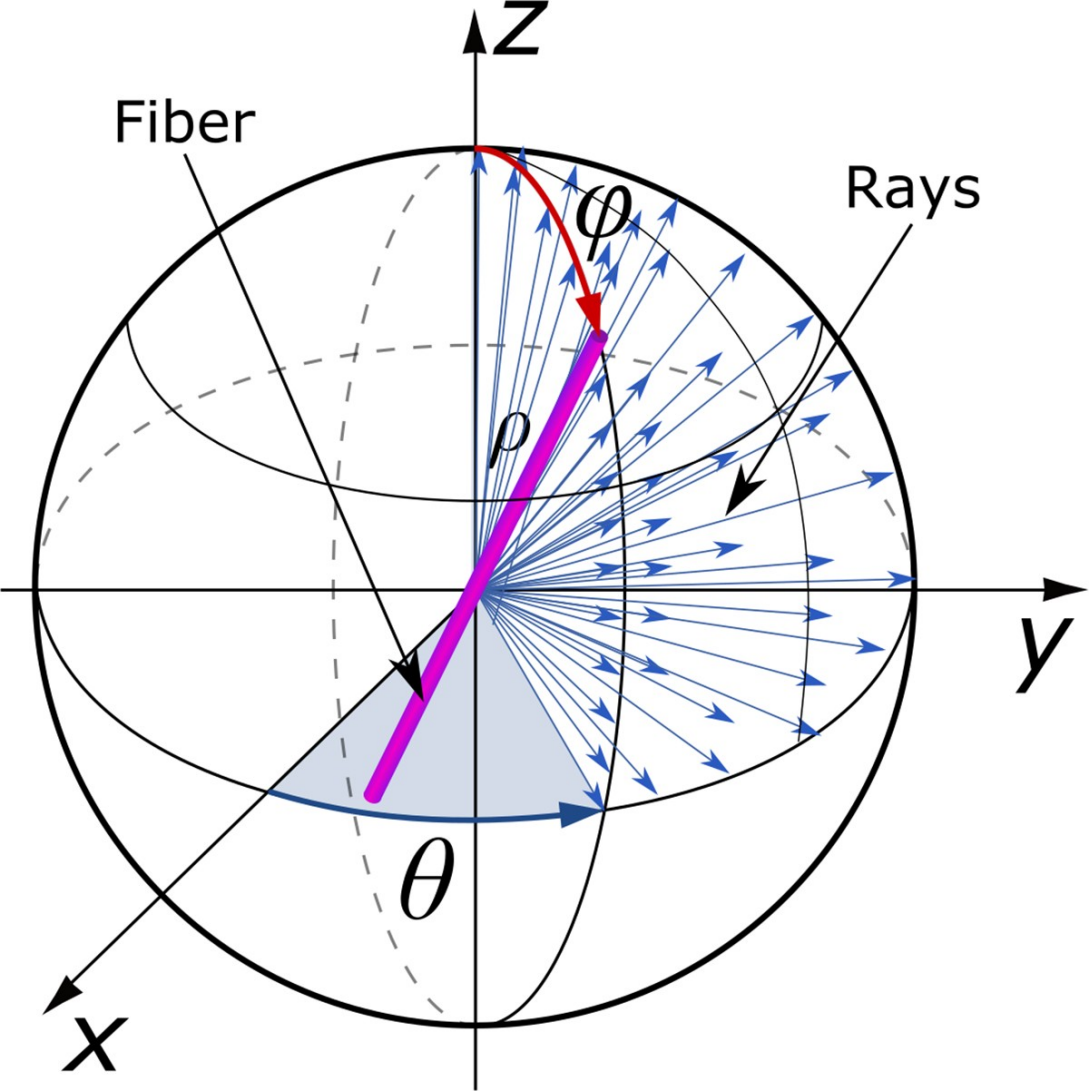
The representation of a fiber in the spherical coordinate system with the rays casted from the origin.

The fiber can be modeled with a cylinder of length ρ with the center of mass placed at the origin of the spherical coordinate system and oriented along the Z-axis (Fig 1). The orientation of the fiber is defined by θ and φ angles determining tilts in different projection planes. The φ angle is the elevation, which represents an inclination relatively the Z-axis and the θ angle is the azimuth, specifying orientation in the XY-plane. We assume that angles vary from 0° to 90° for elevation and from -90° to 90° for azimuth, and both orientation components are 0° when the fiber is co-aligned with the Z-axis.

The proposed method for orientation estimation is based on the concept of emitting a ray in a direction represented by *θ* and *φ* angles at a point of a 3D volume and calculating a ray-sum denoted as:

$$R(x,y,z,\theta ,\varphi )=\int_{L}V(l\;\sin\;\theta \;\cos\;\varphi +x,l\;\sin\;\theta \;\sin\;\varphi +y,l\;\cos\;\theta +z)dl$$ (1)

where, *V* is a 3D volume, (*x,y,z*) is a point within this volume subjected to orientation estimation, *θ* and *φ* are angles describing a direction of an emitted ray, *L* is a length of a ray transmission path in a spherical coordinate system centered at the point that is being estimated. The orientation at this point can be determined using its neighborhood by emitting rays in directions specified by the angular scanning ranges and computing ray-sums. The fiber direction (*θ,φ*) at the point (*x,y,z*) is found by the ray having a maximal ray-sum:

$$R^{\prime }(x,y,z)=\underset{\theta ,\varphi }{\mathrm{argmax}}\;R(x,y,z,\theta ,\varphi )$$ (2)

where, *R*′(*x,y,z*) is the primary orientation, which can be solved numerically by searching through the angular scanning ranges of *θ* and *φ*. Carrying out such operation at every point of *V* creates a huge computational burden and cannot be done in a reasonable amount of time using standard multi-CPU implementations when the size of *V* exceeds several gigabytes. The propagation distance of the rays is limited by a sphere with a radius of *L*/2, centered at the point (*x,y,z*). The tradeoff between the accuracy and the computation time can be achieved by adjusting of the radius of restricting sphere, the limits and the step of the angular scanning ranges.

The proposed approach allows for more accurate orientation estimation of straight fibers since it accumulates values at points along a ray transmission path. The primary orientation is determined by a ray with the highest accumulated sum of values at points constituting the medial axis of the structure. Whereas, the tensor-based approach estimates mean orientation by taking into account all structures within the 3D local window.

### Implementation in pseudo-code

**Fig 2:**
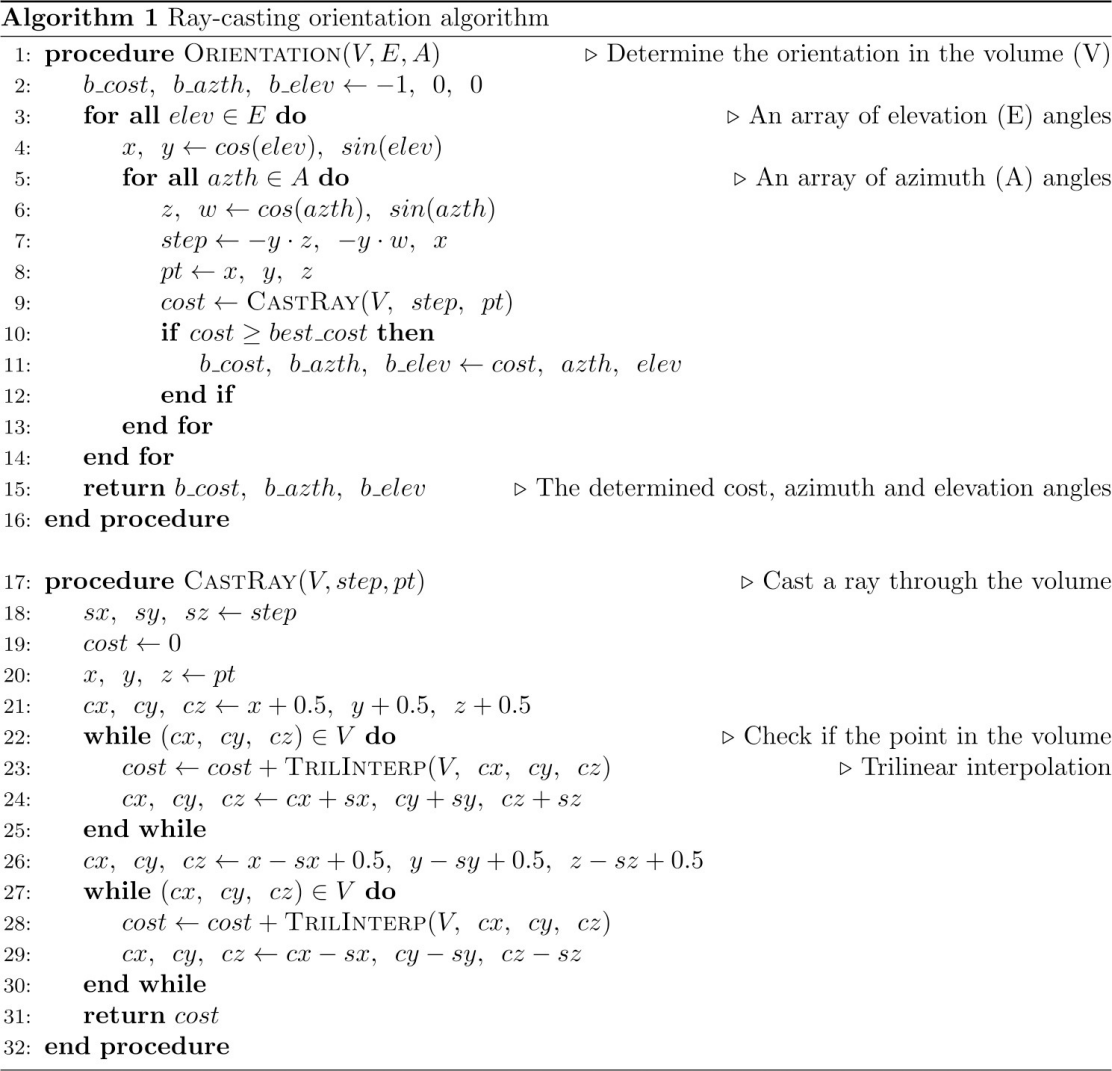
Alg 1—The pseudo-code of the ray-casting method.

The GPU has hundreds of times more cores than CPU, which enables it to perform massively parallel computations. In comparison to sophisticated CPU cores, GPU ones are much more straightforward. They have tiny memory cache levels and aimed at tasks involving as less as possible branching operations. The pseudo-code of the proposed method presented in Alg 1 (Fig 2) was implemented for GPU using the CUDA toolkit provided by NVIDIA. This allowed us to run the method at a vast number of data points at the same time, distributing computation over all the GPU cores, which significantly reduces the total execution time. Moreover, since the proposed approach is based solely on control flow operators, such as loops and conditions, we employed the open-source Numba package [54] for Python language to implement the CPU version. This package enables dynamic hardware-specific vectorization and optimization of code, thereby maximizing the efficiency of using a specific CPU. The algorithm is split into two procedures: the estimation of the structure orientation in the volume restricted by the 3D local window and casting of a ray through the volume at the specified azimuth and elevation angle. It returns the azimuth and elevation angle of the ray, which accumulated the maximum sum of intensities by passing through the volume.

**Fig 3:**
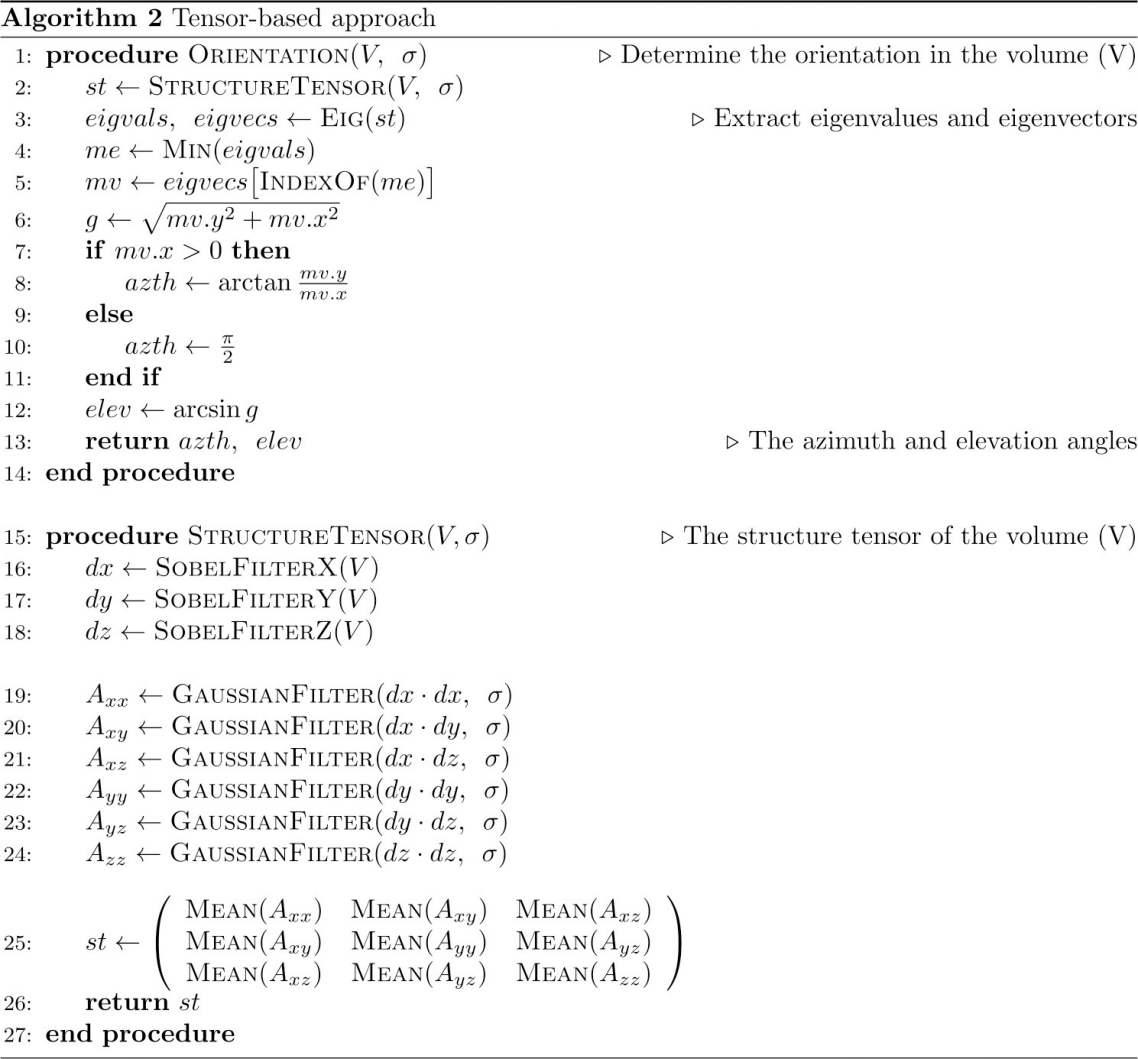
Alg 2—The pseudo-code of the tensor-based approach.

The tensor-based approach [29] is presented in Alg 2 (Fig 3), and it consists of the procedure for calculating the second-order structure tensor and the extraction of eigenvalues and eigenvectors from the obtained covariance matrix. This approach was successfully implemented and parallelized for CPU and GPU, thanks to the open-source TensorFlow package [55] providing computation routines for deriving the eigenvalues and eigenvectors for both hardware platforms. We have implemented our own version because the software package providing this approach does not allow measure the execution time of subroutines and smooth integration into an analysis workflow.

The implementations of these approaches for CPU and GPU platforms are presented in the Quanfima package [56].

### Synthetic data generation

We have generated a synthetic dataset of size 512x512x512 voxels using the algorithm presented in [56] to validate the proposed method and compare it to the tensor-based approach. This dataset is composed of 70 fibers of radius from 3 to 20 pixels with a gap between them from 3 to 10 pixels, oriented in a range from -90° to 90° of azimuth and from 0° to 90° of elevation angle components. Afterward, it was contaminated with the additive Gaussian noise with different the standard deviation (σ_agn_) of 0.5, 1.0, and 1.5, and subsequently smeared with the Gaussian filter of 1.0 and 2.0 sigma (σ_smooth_) values.

### Experimental dataset

The biodegradable polycaprolactone (PCL) 3D scaffolds of a randomly oriented and a well-aligned structure were fabricated using the electrospinning technique [57], which requires a lot of time to find proper fabrication parameters and materials to produce a scaffold with desirable properties [58]. The internal composition of the fabricated scaffolds was measured with the help of a high-resolution μCT setup built on a base of the micro-imaging station placed at a bending magnet source at the Institute for Photon Science and Synchrotron Radiation of the Karlsruhe Institute of Technology (KIT, Karlsruhe, Germany) [59]. The imaging setup employed for the experiment used a monochromatic beam with energy of 12 keV, a 200-μm-thick Lu_3_Al_5_O_12_ scintillator, a 5.5 megapixels sCMOS Camera (6.5 μm physical pixel size), and a BAM macroscope providing magnification of 3.6 times, which results in a spatial resolution of 1.8 μm with a field of view of 4.6x3.9 mm^2^. During data acquisition, the sample mounted at the stage was rotated around the vertical axis for 360° with a step of 0.24° and exposed for 1 sec with X-rays. After that, the acquired data were reconstructed into 3D volumes of size 2016x2016x2016 voxels using the filtered back-projection algorithm [60].

### Analysis workflow

The workflow was implemented in the following stages: the pre-processing stage uses the 3D median filter of size 3x3x3 voxels, the segmentation stage employs the Otsu thresholding algorithm [61] to obtain the binary data, the middle axis extraction was performed with the algorithm from [51], and at the analysis stage both methods were estimated in the specified 3D local window. The whole workflow was implemented and performed on CPUs, except the orientation analysis methods, which were implemented and executed on both CPUs and GPU.

### Benchmarking setup

The performance evaluation was done at a computer operating under 64-bit Ubuntu 16.04 and equipped with Intel Xeon E5-4660 v4 processor, NVIDIA Tesla T4 16GB graphical adapter and 60 GB of random-access memory. The data were located at a corporate storage and were accessed via a high-speed network of 320 MB/s.

## Performance analysis

**Fig 4:**
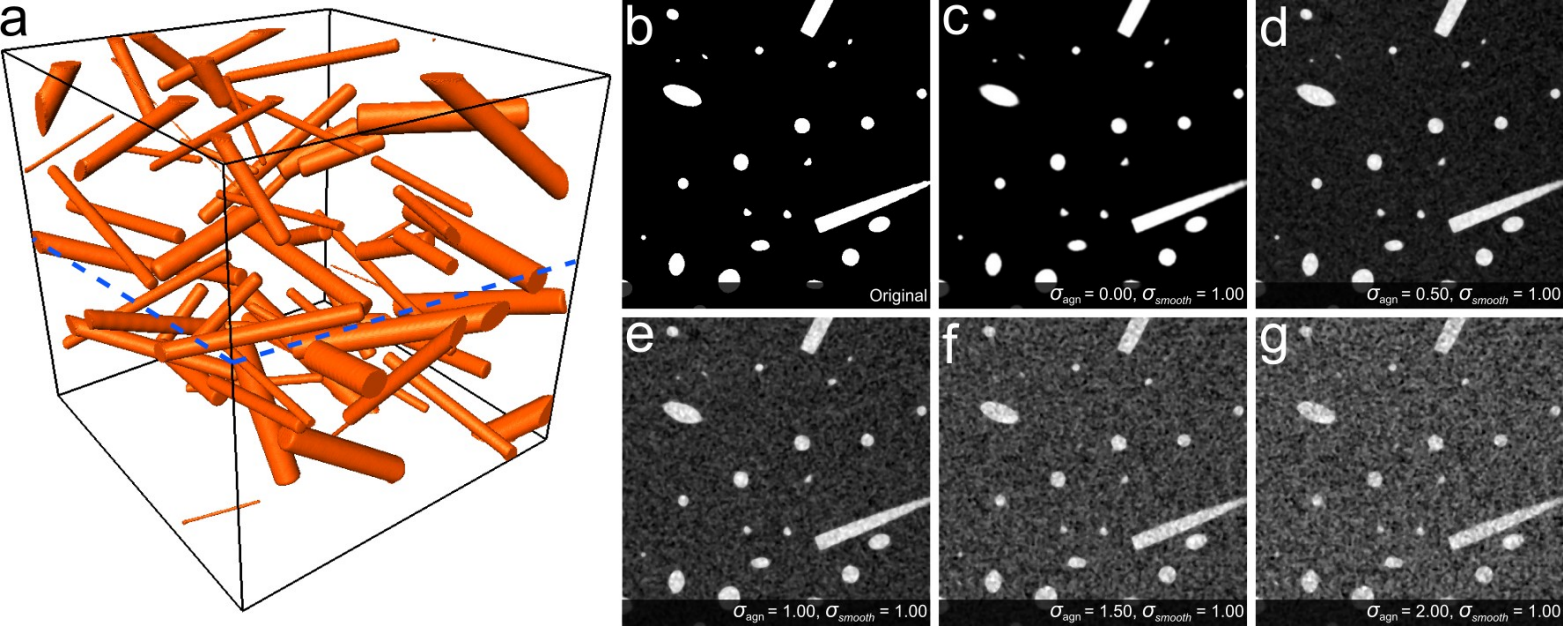
The synthetic dataset (a) with noised slices extracted from the central XY-plane of the contaminated dataset (b-g) (marked with the blue dashed line).

The proposed method and the tensor-based approach were integrated into the described workflow to quantify the orientation of the generated dataset (Fig 4A). The validation procedure is composed of several phases. At the first phase, the noiseless dataset is estimated by each method with varying a window size from 4 to 44 pixels. Then, the window size providing the best accuracy is selected for further calculations. In the next phase, the fixed window size is used in the processing of the generated dataset to observe the behavior and limitations of each method in specific conditions. After that, the throughput of each method was estimated at different scales of the same dataset.

### Accuracy evaluation

**Fig 5:**
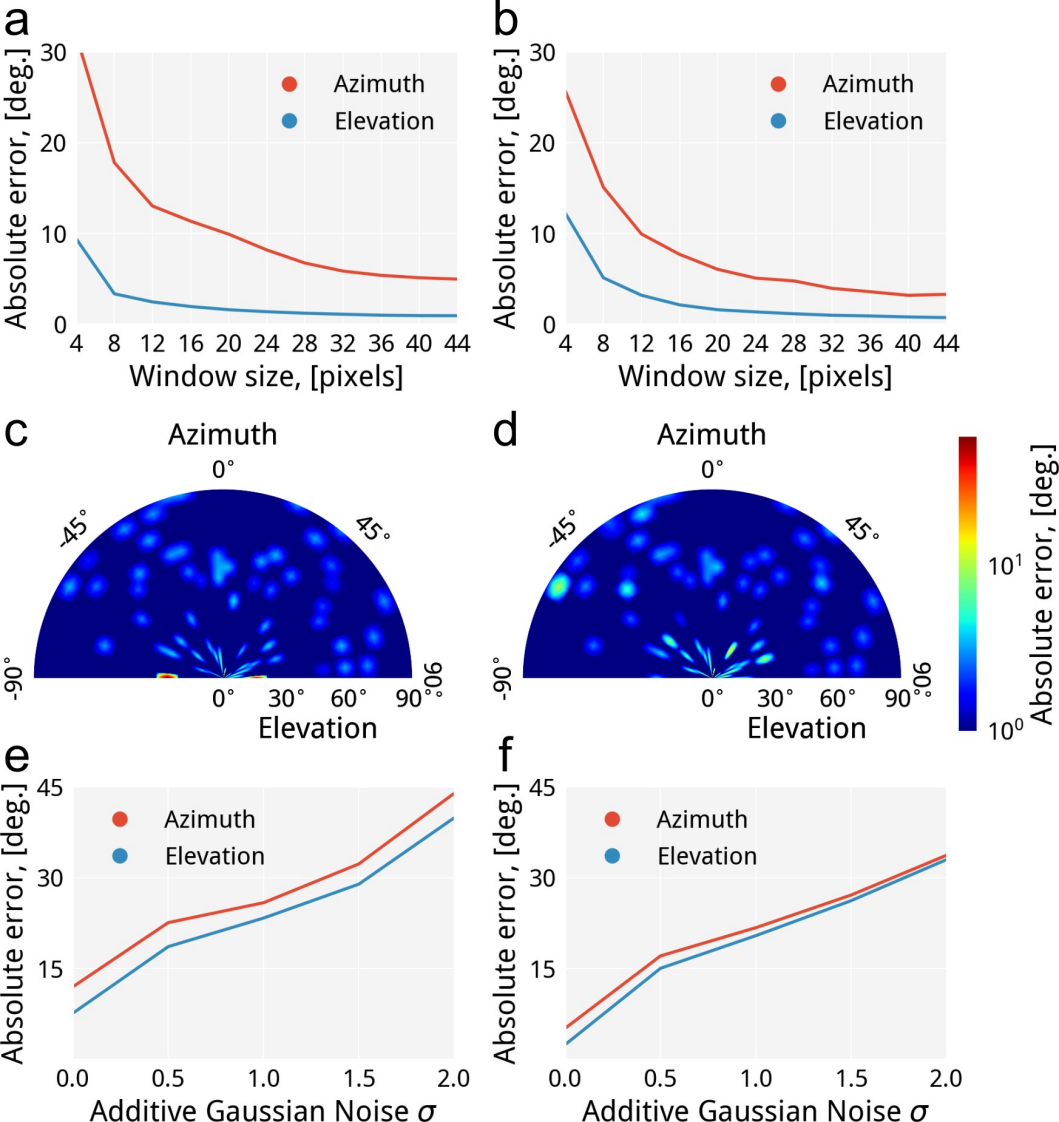
The comparative accuracy analysis of the tensor-based approach (left) and the proposed method (right): a,b) the absolute error of orientation quantification of the noiseless dataset to determine the optimal window size; c,d) the evaluation of the sum of absolute errors to spot error-prone regions on the noiseless dataset; e,f) the behavior of the absolute error while increasing σ_agn_ for the dataset and keeping a window size of 33 pixels.

The methods have a similar behavior of elevation component errors (Fig 5A and 5B), which rapidly fall from 4 to 20 pixels of window size and then do not change much. The results presented in Fig 5A for the tensor-based method show that the sum of absolute angular errors of the azimuth component rapidly falls from 4 to 28 and starts slowly decreasing from 32 pixels of window size. While the azimuthal error of the proposed method in Fig 5B quickly falls from 4 to 24 and then slightly varies. Since the sums of absolute errors of both methods start slowly decreasing from a certain window size, we chose the optimal window size is 34 pixels for the tensor-based approach and 32 pixels in a case of the proposed method. The optimal window sizes were then averaged to unify the parameters of both methods for the further analysis, which results in 33 pixels of window size. Given that the synthetic dataset has radii of fibers in a range from 3 to 20 pixels, with the average fiber radius of approximately 9.5 pixels, then 33/9.5×2 yields the 1.73 factor explaining the relationship between the optimal window size and the fiber radius. The absolute errors produced by the tensor-based approach and the proposed method using the optimal window size are (5.6°±24.29°, 1.03°±0.67°) and (3.75°±8.97°, 0.93°±1.25°) correspondingly, where the values in the parentheses are azimuth and elevation components.

Then, we analyzed which angular ranges are the most error-prone for each method while keeping the optimal value of the window size. The polar heatmaps depicted in Fig 5C and 5D use a logarithmic scale to present the errors calculated as the sum of the averaged azimuth and elevation errors within angular ranges from -90° to 90° and from 0° to 90° for azimuth and elevation correspondingly, with a step of 5° for both. It can be seen from the Fig 5C that most errors of the tensor-based approach are uniformly distributed over the heatmap, except the strong error peaks around (88°, 25°), (-88°, 18°) locations of azimuth and elevation components correspondingly. The proposed method has a comparable distribution of errors (Fig 5D) to its competitor and has weak error peaks around (-60°, 85°), (-50°, 26°), (25°, 16°) and (70°, 26°) locations of azimuth and elevation components correspondingly. The angular error increases towards angles 0°, 90°, and -90° because at these orientation angles tend to be incorrectly determined due to the limited spatial resolution governed by the window size, which in turn is restricted by the proximity of fibers. Thus, the optimal window size should maximize the spatial resolution and minimize capturing neighbor fibers in the window. In a case of tightly packed fibers, the optimal window size will be equal to the diameter of fibers, which is not sufficient to resolve a complete angular range.

Afterward, we run the methods over the synthetic dataset contaminated with σ_agn_ of 0.0, 0.5, 1.0, 1.5, 2.0 and σ_smooth_ of 1.0 to estimate the robustness (Fig 4B–4G). The results presented in Table 1 have shown that the proposed ray-casting approach is more accurate for both azimuth and elevation components for every configuration of noise (Fig 5E and 5F). The angular error almost linearly changes for the proposed method from 0.5 to 2.0 of σ_agn_. However, it non-linearly varies in the same range for the tensor-based approach. This is due to the fact that the segmentation stage produces the over-segmented binary data because of the imposed noise, and subsequently, the extracted skeleton will be significantly distorted, and many wrong locations of orientation estimation are produced.

**Table 1:** The absolute error of methods and the absolute error differences between the tensor-based and the ray-casting approach estimated for the synthetic dataset contaminated with varying σ_agn_.

σ_agn_	Ray-casting approach		Tensor-based approach		Difference of Tensor-based from Ray-casting approach	
	Azimuth [deg.]	Elevation [deg.]	Azimuth [deg.]	Elevation [deg.]	Azimuth [deg.]	Elevation [deg.]
**0.0**	5.11	2.41	11.95	7.59	6.84	5.18
**0.5**	17.05	15.01	22.55	18.59	5.5	3.58
**1.0**	21.71	20.39	25.84	23.30	4.13	2.91
**1.5**	27.11	26.18	32.25	28.91	5.14	2.73
**2.0**	33.67	32.96	43.91	39.86	10.24	6.9

This validation procedure has shown that the proposed method provides higher accuracy than the tensor-based approach for the same dataset over different validation scenarios.

### Throughput evaluation

We analytically estimated the complexity of each method in terms of the required number of arithmetical operations, where N_θ_ and N_φ_ denote the number of azimuth and elevation angles, correspondingly, N_W_ indicates the side length of a 3D local window in voxels, and N_d_ is the size of the square matrix (in our case 3x3 covariance matrix calculated from a 3D local window). It showed that the proposed method requires approximately (N_θ_×N_φ_) × (N_W_^2^+N_W_^2^)^1/2^ arithmetic operations if we assume that each ray has a fixed length of (N_W_^2^+N_W_^2^)^1/2^ and we emit (N_θ_×N_φ_) rays in all possible directions in the 3D local window, thus the computational burden highly depends on the number of emitted rays. Whereas, the tensor-based approach requires N_W_^2^×(10/3×N_d_^3^+N_d_^2^)+(6×N_d_^2^+N_d_) arithmetic operations. The number of required operations was numerically estimated by substituting the variables with corresponding values used in the study, where N_W_ = 33, N_d_ = 3, N_φ_ = 90 and N_θ_ = 180. This showed that the proposed method requires approximately 30×10^6^ arithmetic operations, while the tensor-based method roughly 10^5^. Therefore, the throughput of the sequential computation of the proposed method theoretically is more than an order of magnitude less than for the tensor-based approach. However, this issue can be overcome with help of CPU-specific code vectorizing optimizations and GPUs which are aimed at massive parallelization of fine-grained tasks.

**Fig 6:**
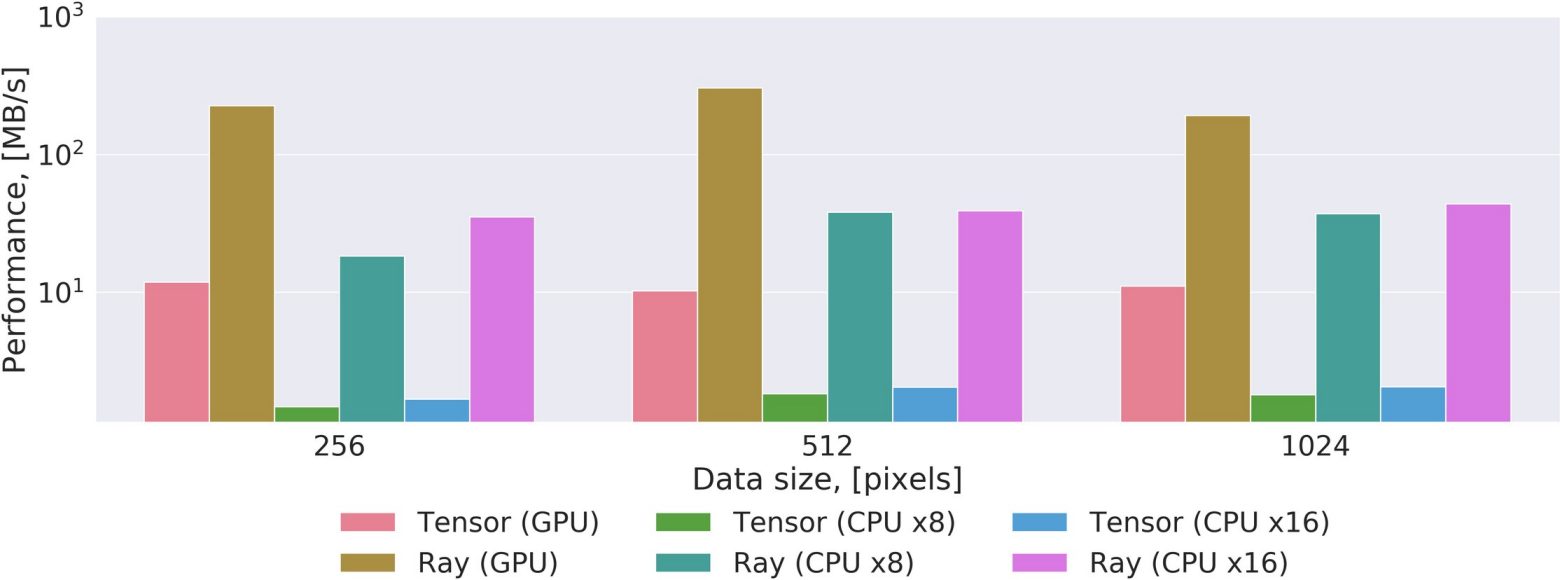
The throughput evaluation of the proposed method and the tensor-based approach over different data sizes for various computation environments.

The performance of the proposed and the tensor-based approach was experimentally evaluated over the synthetic dataset at different scales. We implemented versions of the algorithms for CPU and GPU to compare their throughput in conditions of varying the computational environment and the sizes of the dataset. The results of the evaluation are presented in Fig 6 and Table 2.

**Table 2:** The results of throughput evaluation of the proposed method and the tensor-based approach for various data sizes and computation environments.

Data size (pixels)	Ray-casting approach (MB/s)			Tensor-based approach (MB/s)		
	GPU	CPU x8	CPU x16	GPU	CPU x8	CPU x16
**256**	227.03	18.33	35.41	11.90	1.47	1.68
**512**	304.18	38.26	39.25	10.25	1.84	2.05
**1024**	192.66	37.34	43.92	11.07	1.80	2.07

The CPU version of the proposed method is 12–20 times faster than the tensor-based approach in the same conditions, and this tendency is preserved for all cases of computations involving CPUs. The throughput of the proposed method on CPUs is always higher than the tensor-based method because it is based on control flow operators and trivial memory access patterns allowing for hardware-specific optimizations, whereas the method competitor is locked to the specific implementation of the eigen-decomposition algorithm. In the case of the GPU version, the proposed method outruns in 17–30 times the tensor-based approach, because the latter cannot be easily parallelized due to the eigen-decomposition algorithm. Thus, the undertaken experiment showed the high suitability of the proposed method for implementation on both CPU and GPU and its superiority over the tensor-based approach in all considered evaluation scenarios.

## Application

We have performed a 3D orientation analysis of microfibrous scaffolds made from biodegradable PCL polymer with aligned and randomly oriented fiber structure using the presented method. Such scaffolds are widely applied in regeneration medicine as they serve as constructions which imitate an extracellular matrix of native bone tissue [62].

The experimental datasets were cropped to 600x600x450 voxels, corresponding to 1080x1080x810 μm^3^, and then subjected to the previously described workflow to pre-process each dataset before the analysis. At first, each slice of the reconstructed 3D volumes was filtered with a median filter of radius 1.8 μm. Then, each filtered slice was segmented with the Otsu thresholding algorithm [61] to provide slices where only fibers have non-zero values. Next, the medial axis was extracted from the segmented data using the axis thinning algorithm [51]. The optimal window size was selected based on the averaged expected fiber diameter of 33 μm corresponding to 18.3 pixels given the spatial resolution of 1.8 μm and taking into account the previously calculated factor of 1.73, it yields the optimal window size of 32 pixels. Then, the orientation analysis was performed over the extracted central axis by estimating the proposed algorithm in the neighborhood 32x32x32 voxels of every voxel belonging to the central axis. Even though we estimated the optimal window size, the diameter fluctuations of the fibers will not significantly affect the orientation analysis results, since the orientation is estimated over the extracted medial axis of the fibers, thus only the spatial proximity of fibers may have an impact.

**Fig 7:**
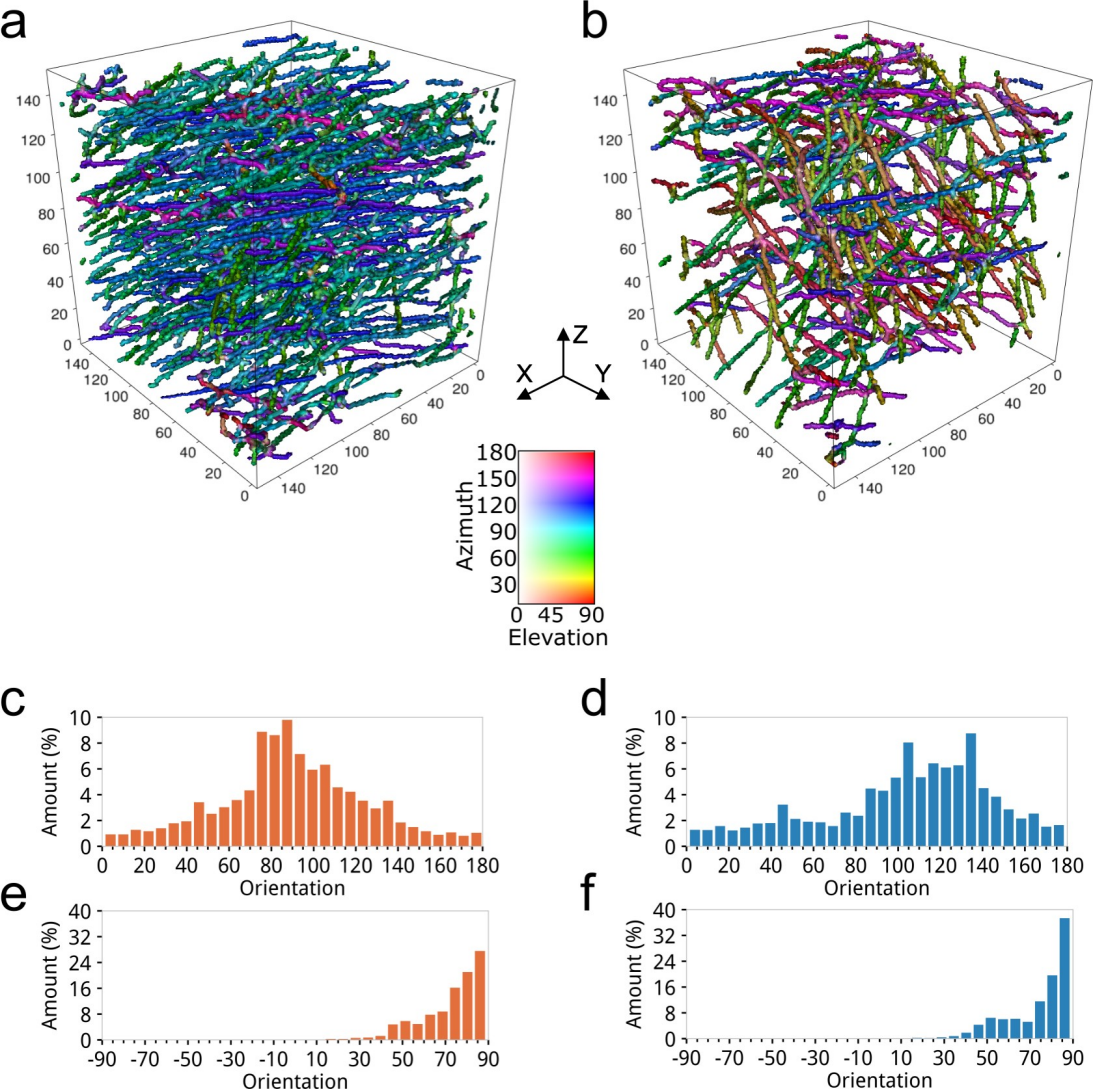
The 3D orientation analysis results produced by the proposed algorithm for estimating orientation on the datasets of PCL scaffolds acquired at the synchrotron-based μCT imaging setup: a,b) the 3D visualization of the color-coded fiber orientation datasets of the scaffolds with well-aligned and randomly oriented structure; c,d) the azimuthal orientation histograms; e,f) the elevation orientation histograms of fibers for well-aligned and randomly oriented cases.

The 3D orientation analysis presented in Fig 7 has shown that in the azimuthal direction, the samples with a well-aligned structure (Fig 7C) demonstrate prevalent fiber orientation in the range from 70° to 100°. Whereas, the scaffolds with a randomly oriented structure (Fig 7D) have directionality of fibers distributed in the range from 80° to 160° due to the type of rotating collector. However, all samples demonstrate similar results in the elevation direction (Fig 7E and 7F)), which concentrated in the range from 50° to 90° because fibers were deposited layer-by-layer during the electrospinning process.

The results of 3D orientation analysis allow immediately distinguish between randomly oriented (Fig 7B) and well-aligned (Fig 7A) fibers by a color coding, where the color of the same shade represents a similar direction. As it may be observed, fibers of the sample with a well-aligned structure change their preferred orientation depending on the height level of the sample. It could be due to a difference in the layer deposition during electrospinning, where each layer is deposited individually. While in the sample with a randomly oriented structure, fibers are chaotically distributed in the sample volume, and some of them are linked in depth to other layers. Such a detailed 3D orientation analysis allows for the controlled fabrication process to produce scaffolds with desirable fiber properties [58].

## Discussion

In recent years a vast number of methods aimed at orientation analysis of structures in datasets of diverse modalities were presented. Initially, every method was developed to answer a specific question about a particular dataset. However, late due to the inherent generality, some of them were successfully applied to other problems, such methods as analysis of the Fourier spectrum or the tensor-based approach. The latter has been compared to the proposed method in this study. The proposed method outperformed the tensor-based approach in terms of accuracy, and due to inherent parallelizability, it can be efficiently implemented for GPU to improve the execution time drastically. Moreover, the processing speed can even be further improved by shrinking the scanning angular ranges. The coarse-to-fine approach can be used to create the multi-level pyramid of the dataset. Then, starting from the top level, the orientation angles are calculated at each point and propagated to the next level. The orientation is recalculated for the new level at each point, taking into account the angular values at the previous level and some confidence intervals to mitigate the accuracy errors. The process repeats until it reaches the latest level, where the initially large scanning ranges are significantly shrunk. The described changes will be introduced in the further revisions. Despite the proposed method outperformed the tensor-based approach, it cannot completely replace it. Our method is mainly oriented to the analysis of the datasets with straight and clearly separable structures because it relies on intensity accumulation along a ray path. While the method competitor is more suitable to analyze stuck together or bent structures by quantifying average orientations in the regions-of-interests.

## Conclusions

We presented a new method based on the ray-casting concept aimed at quantification of structures orientation in datasets from various sources. The method was validated on a synthetic ground-truth dataset. It provides higher accuracy than the popular tensor-based approach, and it has an inherent potential for the efficient implementation for GPUs. It was successfully applied for the orientation analysis of microfibrous scaffolds with aligned and randomly oriented fiber structure. In future work, the method will be improved regarding speed by introducing the coarse-to-fine strategy to reduce the scanning angular ranges.
